# Biocides as Biomedicines against Foodborne Pathogenic Bacteria

**DOI:** 10.3390/biomedicines10020379

**Published:** 2022-02-04

**Authors:** Eugenia Butucel, Igori Balta, Mirela Ahmadi, Gabi Dumitrescu, Florica Morariu, Ioan Pet, Lavinia Stef, Nicolae Corcionivoschi

**Affiliations:** 1 Bacteriology Branch, Veterinary Sciences Division, Agri-Food and Biosciences Institute, Belfast BT4 3SD, UK; eugenia.butucel@afbini.gov.uk (E.B.); igor.balta@afbini.gov.uk (I.B.); 2 Faculty of Bioengineering of Animal Resources, Banat University of Animal Sciences and Veterinary Medicine—King Michael I of Romania, 300645 Timisoara, Romania; mirelaahmadi@usab-tm.ro (M.A.); gdumitrescu@animalsci-tm.ro (G.D.); floricamorariu@animalsci-tm.ro (F.M.); ioanpet@eurofins.com (I.P.); 3 Faculty of Animal Science and Biotechnologies, University of Agricultural Sciences and Veterinary Medicine, 400372 Cluj-Napoca, Romania

**Keywords:** biocides, biomedicines, disinfection, antibiotic–biocide cross-resistance, foodborne pathogens

## Abstract

Biocides are currently considered the first line of defense against foodborne pathogens in hospitals or food processing facilities due to the versatility and efficiency of their chemical active ingredients. Understanding the biological mechanisms responsible for their increased efficiency, especially when used against foodborne pathogens on contaminated surfaces and materials, represents an essential first step in the implementation of efficient strategies for disinfection as choosing an unsuitable product can lead to antibiocide resistance or antibiotic–biocide cross-resistance. This review describes these biological mechanisms for the most common foodborne pathogens and focuses mainly on the antipathogen effect, highlighting the latest developments based on in vitro and in vivo studies. We focus on biocides with inhibitory effects against foodborne bacteria (e.g., *Escherichia* spp., *Klebsiella* spp., *Staphylococcus* spp., *Listeria* spp., *Campylobacter* spp.), aiming to understand their biological mechanisms of action by looking at the most recent scientific evidence in the field.

## 1. Introduction

Foodborne pathogens are of major importance to public health due to their ability to cause severe diseases and death in humans, emphasizing the need for constant monitoring and prevention [1]. Because of their ability to proliferate in food and water, foodborne infectious agents are a major concern for the food industry, consumers, and medical and veterinary practices [2]. Controlling their proliferation can be impaired by the acquisition of resistance mechanisms, via gene mutations or plasmid-encoded resistance, leading to the so-called targeted resistance, as unlike antibiotics biocides are able to directly target biological structures or mechanisms within the bacterial cell (e.g., membrane permeability or biocide efflux) [3]. The increased ability of pathogen proliferation within the host and the acquisition of resistance to biocides are two factors that are increasingly associated with nosocomial infections, representing a significant burden for the global health systems. The effects include high mortality and morbidity rates, high treatment costs, diagnostic uncertainties, and a lack of confidence in medicine [4]. According to the World Health Organization, the recently announced group of emerging pathogenic agents, ESKAPE, includes both Gram-positive and Gram-negative microorganisms (e.g., *Staphylococcus aureus*, *Klebsiella pneumoniae*, *Pseudomonas aeruginosa*) which are the common causes of life-threatening nosocomial infections in humans [5,6]. A major reservoir of such pathogens is the farming industry, where they have the capacity to form biofilm and become biocidal- and antibiotic-resistant.

The availability of efficient disinfection procedures is a critical element of the entire biosecurity programs designed to prevent and eliminate a wide diversity of life-threatening pathogens in agriculture, healthcare, and food industry facilities. These antipathogen chemicals, displaying bactericidal or bacteriostatic effects, are called biocides. They can be defined as broad-spectrum anti-infective application products, including disinfectants, preservatives, and agents to prevent microbial growth [7]. Biocides are classified as substances and preparations containing one or more active blends of substances to destroy, prevent the development of, or control any harmful microorganism via chemical or biological activities [8,9]. Recent developments allowed the inclusion of biocidal chemicals as composites in various articles, such as surgical scrubs, medical devices, mouthwashes, wound dressings, and antibacterial soaps [10]. 

By definition, most biocides can be toxic to some form of living organisms, with toxicity manifested as direct cytotoxicity, systemic direct or indirect toxicity, and allergenic activity leading to oxidation or solubilization of the lipids in the cell wall. The mechanism of biological action differs depending on the chemical substance, the presence (synergism) or absence of other chemicals, and the type of microorganism to be eradicated or attenuated. Some of the possible examples of mechanisms of action of biocidal antimicrobial substances are considered to cause oxidation of lipids from the membrane surface, which subsequently causes lesions and disturbance of transport and enzymatic activity [11,12,13].

Numerous reports mention the possibility of antibiotic–biocide cross-resistance in pathogenic bacteria [14,15,16,17]. This emphasizes the fact that indiscriminate use of these substances and contamination of both water and soil environments are leading to uncontrolled release of biocides into the environment and the potential of bacterial resistance occurrence [18]. For example, the attachment of bacteria to different surfaces (e.g., industrial, hospitals, and medical devices) leads to biofilm formation, posing a serious threat to human health or to biosecurity in various industries. Ineffective disinfection of surfaces and improper use of biocides can lead to the survival of bacteria and viruses, often contributing to the transmission of infectious agents [19,20]. The biocidal resistance of planktonic cells has been attributed to gene mutations or changes in the cellular envelope, activation of the efflux pump, or expression of enzymes that cause biocide degradation [21]. 

Consequently, the developed bacterial tolerances initiated a search for biocides derived from natural sources, including plants, peptides, enzymes, or even nanotechnology fabrication of novel biocidal products [22]. This review will successively consider the different compounds used as biocides and their mechanisms of action, before successively presenting their effects on the main emerging pathogens. Our approach in this review was to bring together clusters of scientific data on the efficient applications of biocides for sanitation and disinfection purposes to combat bacterial foodborne pathogens in different settings.

## 2. Biocides

### 2.1. Metal-Based Biocides

Metal-based disinfectants (e.g., heavy metals) are often used as chemotherapeutic agents to prevent infections in humans and animals [23]. Among these, metal cations and oxyanions are well known for their antimicrobial effects, especially for their toxicity towards most biofilm-forming bacteria [24]. As for all the other biocides, the ability of bacteria to develop resistance to metals must be one of the main priorities during the developmental process as they have the ability to co-select for antibiotic-resistant bacteria which could reduce their ability to prevent or eradicate biofilms [25]. This mechanism of acquired resistance is involved in the transfer of antibiotic resistance genes, especially if the metal-biocide genes are located on the same plasmid [26]. One example is pyridoxine, which was proven to have an antibiofilm effect when tested against *S. aureus* and *S. epidermidis* biofilms, leading to complete eradication at concentrations of 64 and 16 μg/mL [27]. Another example of efficient antibiofilm metal-based biocides is silver nanoparticles, which in combination with ammonium salts have been shown to eradicate bacterial biofilms [28]. In this case, the ammonium salts play the role of a facilitator of drug diffusion into the biofilm [29], a role also emphasized in a more recent study [30]. The biology and mechanisms of action of metal-based biocides were very recently reviewed, and their role in treating bacterial infections was emphasized especially in the case of antibiotic resistant bacteria [31]. Further details on their biological mechanisms of actions are included in Table 1, Table 2, Table 3 and Table 4.

### 2.2. Aldehydes, Alcohols, Phenols, and Bisphenols

Formaldehyde, occasionally used in veterinary hygiene, interacts with bacterial proteins, DNA, and RNA by crosslinking free amino groups specifically at low temperature, which is considered an efficient method for the disinfection and sterilization of surgical equipment. The interaction is associated with the alkylation of sulfhydryl and amino groups from proteins and the alkylation of the ring-nitrogen atoms belonging to purine nucleotide bases which inhibit transport mechanisms [32]. Phenolic compounds, on the other hand, have a wider spectrum of activity against bacteria and also fungi and viruses. One of the most common bisphenols is triclosan (TCS), a compound used in the preparation of antiseptic soaps and widely used in industries, being incorporated into various products such as toys, deodorants, toothpaste, and cosmetics [33,34]. The bactericidal mechanism of action of phenols and bisphenols occurs via the disruption of the cytoplasmic membrane, which leads to a rapid release of intracellular components. TCS has a specific target, enoyl-ACP reductase in bacteria, encoded by the *fabI* gene, involved in fatty acid synthesis [35]. Phenol (carbolic acid) is one of the old and classical antiseptic agents with a prominent fungicidal and bactericidal activity that can be improved with the addition of EDTA and warmer temperatures [32]. On the other hand, low temperatures, lipids, and higher alkalinity can diminish and considerably affect its biocidal properties [9]. On the other hand, low temperatures, lipids, and higher alkalinity can diminish and considerably affect its biocidal properties [9].

### 2.3. Halogens, Peroxides, and Organic Acids

Halogenic compounds (e.g., hypochlorite; iodophor; iodine-, bromine-, and chlorine-containing compounds) can oxidize proteins within a cell, penetrate the cellular membrane, and cause the interruption of the oxidative phosphorylation systems [32,36]. The peroxide-based biocides (e.g., peroxyacetic acid and hydrogen peroxide) on the other hand are mainly used for disinfection of surfaces (metallic, glass, or plastic), with high efficacy against bacterial and viral pathogens [37]. Organic acids (e.g., lactic, acetic, and citric acids) are also commonly used as biocides by inclusion in food as preservatives and are considered to exhibit antimicrobial action through pH-mediated coagulation of proteins [37]. Recent studies indicated that natural antimicrobial formulations enriched with organic acids demonstrated a strong ability to reduce bacterial attachment to chicken skin and carcasses during the washing process [9,20].

### 2.4. Quaternary Ammonium Compounds (QACs)

Quaternary ammonium compounds (e.g., alkyl trimethylammonium bromides (cetyltrimethylammonium bromide, dialkyl dimethylammonium chloride, and didecyldimethylammonium chloride), benzalkonium chloride, Miramistin, cetylpyridinium chloride, octenidine dihydrochloride, twin/gemini surfactants) are some of the most used disinfectants and antiseptics in different fields, including households, agriculture, industry, and medicine [38]. Some of the antipathogenic roles of QACs include pH modification and the induction of damage of cellular membranes causing disruption [32]. Benzalkonium chloride (BAC) and cetrimide are the two main agents in the QAC group that are active against the coverings contaminated by bacteria, viruses, and fungi. They are normally used to disinfect areas where animals are kept and transported (e.g., floors, walls, and transport vehicles) and are also used to disinfect food-handling areas. In bacteria, the main target for QACs is the cytoplasmic membrane. BAC can bind to anionic sites on the outer surface of bacteria that cause destabilization and solubilization of cells and can disintegrate proteins, enzymes, and nucleic acids [39].

### 2.5. Phytochemicals

Phytochemicals are considered an unexploited source of new biocides [40]. Plant extracts show a promising biocidal potential among the alternative treatments to be investigated, with many advantages over traditional synthetic options. They are environmentally friendly (biodegradable) and have a functional complexity (preventing bacterial resistance transmission). Plants can synthesize many organic compounds classified as primary and secondary metabolites. The possible advantage of these substances is that they express a significantly lower number of side effects when compared to the case of the use of synthetic chemical substances as they undergo detoxification and are poorly absorbed with rapid excretion [41]. New formulations of antimicrobial mixtures, including combinations of organic acids and plant extracts, were proven in in vitro and in vivo studies to have more efficient antimicrobial activity against different pathogens by acting directly or indirectly against their principal virulence factors [22], as we describe below.

## 3. Biological Mechanisms of Action of Biocides

**Figure 1:**
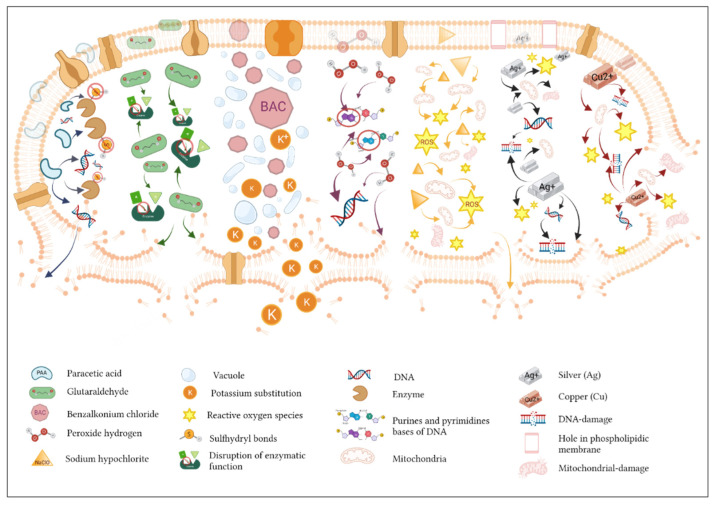
Biocides and their main modes of action. Peracetic acid triggers the oxidation of sulfur and sulfhydryl bonds of enzymes and proteins, causing cell wall disruption leading to lipoprotein blockage in the cytoplasmic membrane and generation of peroxide radicals. Glutaraldehyde favors cross-binding with the amine-presenting lipids of bacterial cells and inhibits the transport of enzymes and functions by inducing a bactericidal effect. Benzalkonium chloride induces destabilization of the membrane permeability and vacuolation, modifies the K^+^ intake, and induces cell surface rupture and extravasation of the cellular content. Hydrogen peroxide enables passive diffusion, penetrates the membrane, and targets the DNA and mainly the purine bases (guanine and adenine). Sodium hypochlorite stimulates ROS formation and cell membrane rupture. Silver (Ag) mainly affects phospholipids and creates holes in the outer membrane by increasing permeability, causing disruption of the breathing cycle that leads to ROS formation and DNA damage. Copper (Cu) is prone to ROS formation, which causes irreversible changes to DNA and inflicts damage to the membrane, causing loss of intracellular components and finally cellular death.

The processes involved in biocide antimicrobial activity include the transport of the antimicrobial agent to the cell surface, adhesion, invasion, diffusion, penetration, and interaction at the target site (Figure 1). Of course, these processes are not instantaneous, and their action in time may differ depending on the biocide agent. The differences also refer to their mode of action, their chemical composition, and the physicochemical properties of each biocide [42]. The colloquial definition of an antimicrobial effect is related to the interaction between an active substance and specific targets of the microbial cell. The critical characteristics of these interactions are the physicochemical properties of the biocide, the morphology of the cells, and the physiological state of the microorganism [43].

Although the mechanisms at the origin of the biocidal effects vary according to the compounds because of their structure, their consequences at the biofilm scale lead to a succession of similar steps. Typical antibacterial events include the following [44]: (i) inhibition of respiration or inhibition of catabolic/anabolic pathways, (ii) destabilization of membrane integrity that leads to the leakage of essential intracellular constituents, (iii) the disruption of the motor force of the transmembrane proton that leads to a decoupling of oxidative phosphorylation and the inhibition of active transport on the membrane, (iv) lysis, (v) interruption of replication, and (vi) coagulation of the intracellular material.

### 3.1. Release-Killing Biocides and Contact-Killing Biocides

“Release-killing” biocides are incorporated on surface coatings in order to allow their constant release into the environment and to kill nearby bacteria. These types of biocidal products may contain antibiotics, metal nanoparticles (e.g., Ag^+^ and Cu^2+^), nitrogen oxides, and other substances. Their mode of action can include inhibition of DNA replication or protein synthesis, destruction of the cell membrane, and the capacity to inactivate metabolic enzymes and deteriorate other important proteins [45,46]. Unlike the release-killing biocides, the “contact-killing biocides” are permanently immobilized by physical or chemical interactions on the surface. Biocides used for this purpose can be assembled from natural biomacromolecules (e.g., chitosan, antibacterial peptides (AMPs)), antibacterial enzymes (AMEs), nylon polymers, and others [47,48,49,50]. These compounds and polymers are based on a strong electrostatic interaction between positively charged groups and negatively charged phospholipids on the bacterial cell membrane, which leads to general disturbances of the lipid bilayer and leakage of proteins and other intracellular components [51]. In addition, some AMEs may catalyze the degradation of polysaccharides across the cell membrane or suppress quorum sensing, triggering bacterial death [52]. For N-halamines, direct transfer of active halogen to bacteria may inhibit their metabolic process [53]. The main difference between the “release-killing” and the “contact-killing” biocides resides in the fact that the latter are considered less efficient in inducing pathogen resistance [54].

### 3.2. Biocide Efficacy

The effectiveness of a disinfection procedure is dependent on the correct application of an effective biocide. However, inefficiency can have multiple explanations, and bactericidal resistance is one example. There are several reasons for the loss of effectiveness in a disinfection procedure that are easy to confuse with acquired resistance, including inefficient application, prolonged application, inefficient contact with the pathogen, and low availability [55]. Biocidal resistance describes the relative insensitivity of a microorganism to a particular treatment under a certain set of conditions [56]. It is usually quantified as the concentration that causes sublethal effects in addition to biocide resistance which can be a natural property of a microorganism or acquired through mutation or the acquisition of self-replicative plasmids [57]. According to the Weibull model, the bacterial population can be divided into subgroups with different death rates (e.g., very sensitive, sensitive, and resistant cells). Interestingly, at higher concentrations the antimicrobial effects are easily induced; however, most bacteria are tolerant at low concentrations, most possibly due to intrinsic defense mechanisms [19].

### 3.3. Inducing the Coresistance and Tolerance of Microorganisms through the Use of Biocides

The excessive use of biocides and antibiotics has a considerable economic and environmental impact, and inappropriate use of more assertive biocides and high doses (as far as overcoming resistance) poses an additional risk to public health [22]. Reduced susceptibility of microorganisms to biocides can be achieved by mutation or acquiring a plasmid or a transposon [58]. Acquired resistance to antibiotics also occurs through mutations or acquiring genetic elements through plasmids and transposons [59]. The acquired resistance can result in bacteria being able to grow in a biocide–antibiotic concentration that increases gradually, leading to the so-called acquired coresistance. However, this temporary resistance to phenotypic adaptation is unlikely to play an important role in determining the long-term survival of bacteria in biocides [55].

As a promising alternative strategy in the control of cross-resistance, the combined chemotherapy of two or more anti-infective compounds (synergism) has been prominently employed to treat complex infections, including respiratory diseases, which often involve viral and bacterial strains [60]. Research is extensively focused on applying chemical substances that act on various bacterial targets, including adhesins, bacterial toxins, and quorum sensing (QS) signaling [61]. The constant administration of anti-infective compounds has raised questions about the positive selection of resilient bacterial strains [62]. The mechanisms involved in these processes are not fully understood and are yet to be elucidated. To date, the mechanism of action was associated to bacterial resistance to anti-infective agents and include membrane adaptation [63]; changes in the expression of enzymes, inducers, and repressor genes; and horizontal transfer of resistance genes [64], including those involved in biofilm formation [65] and QS signaling. In a recent study realized by Sonbol et al. [63], adaptation processes of *E. coli* isolates treated with sublethal concentrations of triclosan were described. A decrease in membrane permeability (outer and inner) and depolarization and an increase in the negative potential was observed. Moreover, triclosan was found to be a substrate for efflux pump expression in bacterial strains, and overexpression in efflux activity was observed in *E. coli* strains [63].

## 4. The Effect of Biocides against Foodborne Pathogenic Bacteria

### 4.1. Escherichia spp.

Several *Escherichia* species are natural residents of the intestinal microbiota, but pathogenic species also cause urinary tract infections and the dysbiosis of the reproductive tract [66]. For example, clinically relevant *E. coli* O157:H7 was involved in serious waterborne and foodborne outbreaks leading to severe human infections of the gastrointestinal tract and hemolytic uremic syndrome [22,67]. As such, access to clean, germ-free water is essential, but water contamination remains a global concern. As a consequence assembling a filter with biocidal properties to purify drinking water could be a way to eliminate pathogenic *E. coli*. Water treatments with semipermeable membrane filters have been advanced significantly in recent years, and techniques may include microfiltration, ultrafiltration, and nanofiltration, providing removal of harmful microorganisms [68]. The advantages of membrane filtration are due to increased efficiency and low energy consumption; moreover, filters are environmentally friendly, compact, and easy to maintain [69]. An example of a cellulosic filter combined with butane tetracarboxylic acid (BTCA) and glutaraldehyde was recently described [13]. The results indicated that glutaraldehyde solution or 1,2,3,4-butanetetracarboxylic acid (BTCA) added to cellulose pretreated with cationic polyacrylamide (C-PAM) increased antimicrobial activity against *E. coli*, an effect apparently caused by glutaraldehyde interaction with the nonprotonated amines from the outer layer of the bacterial cell, leading to disruption of bacterial enzymatic activity.

In planktonic and biofilm states [70], sodium hypochlorite (SH) showed the fastest biocidal action, while NaDCC (chlorine-based biocide) showed the highest antimicrobial rate but with lower efficiency. The biofilm assay demonstrated that such biocides caused logarithmic declines by approximatively > 3.20 log CFU per cm^−2^, possibly due to the production of reactive oxygen species (ROS). The authors demonstrated that neutral electrolyzed oxidizing water (NEOW) displayed the most extended decay time for total chlorine loss (70 days at 5 °C), possibly because it allowed the highest formation of reactive oxygen species (ROS). NEOW also had the most reduced chlorine loss rate (0.013 ppm/min at 5 °C), suggesting this biocide as a suitable alternative to SH biocides, which showed the most increased chlorine loss rate (0.025 ppm/min at 5 °C) [38].

The novel pyridoxine-based quaternary ammonium derivatives from terbinafine [11] had an in vitro antimicrobial activity of 8 μg/mL MIC against *E. coli* (MG1655). The MIC values are caused by a dual antimicrobial molecular mechanism, described as bimodal, which integrates the ability to induce membrane integrity damage and targeting of pyridoxal-dependent enzymes. Other engineered peptidic biocides, synthesized from 45 indolicidin analogs [71], displayed MICs of 2.7 μg/mL for *E. coli* ML-35p, 2.5 μg/mL for *E. coli* M17, and 1.8 μg/mL for *E. coli* 25293. The authors indicated that the possible biocidal mechanism of action occurs due to the disruption of the cell membrane and is mainly conditioned by the amphipathicity and hydrophobicity of indolicidin molecules.

Of the newest disinfectants, the electrochemically activated solutions (ECASs) have attracted significant interest due to their environmentally friendly nature and the persistence of different oxidants with a more elevated disinfection prospect when compared to chlorine-based biocides [72]. The authors of a recent study concluded that ECASs are promising biocides with different chemical reactive oxidative species capable of rendering microorganisms inefficient at optimal concentrations and changing their molecular ionic structure [72]. The inactivation kinetics with different concentrations (1%, 5%, and 10%) indicated that *E. coli* exhibited a maximum 3.50 log CFU/mL reduction and was more vulnerable to ECASs than *Aeromonas* spp.

**Table 1:** Biocides and their impact on *Escherichia coli*.

Biocide	Strain	Concentration	Mechanism/Notes	Gene/Protein	Ref.
MBQ (magnetic biochar/quaternary phosphonium salt)	*E. coli* ATCC 25922	MBC 20 mg/L	Cell wall and membrane penetration; induced vacuolization, loss of cell integrity/permeability, leakage of intracellular components, oxidative stress.	n.i.	[73]
Cellulose foam paper with BTCA and GA	*E. coli* MTCC 40	MIC 1 g/L	BTCA—crosslinking agent. Reduced pH and growth. GA—cross-binding with cell wall amines interfered with the transport and enzymatic activities and bactericidal effect.	n.i.	[13]
Polyvinylidene fluoride membranes with guanidine backbone or sulphonium backbone	*E. coli* ATCC 25922		Bacteria lost structural integrity caused by the existing electrostatic interactions, which led to the leakage of intracellular components and caused their death.	n.i.	[12]
Sodium hypochlorite; chlorine dioxide; neutral electrolyzed oxidizing water; sodium dichloroisocyanurate	*E. coli* CECT 434	MBC 80 and 100 ppm—planktonic cultures; 50 ppm—biofilms	ROS production in the case of SH and NEOW.	n.i.	[38]
KFU-127 (pyridoxine-based quaternary ammonium derivatives of terbinafine 127)	*E. coli* MG1655	MIC 8 µg/mL MBC ×16	Cell membrane damage and membrane potential changes; inhibitory property on pyroxidal-dependent enzymes.	n.i.	[11]
Peptide biocides (45 analogs of antimicrobial peptide indolicidin)	*E. coli* M17; *E. coli* ATCC 252934	MIC 0.9–1.8 µg/mL	Pore-forming agents; the introduction of groups Cl, NO_2_, F into the aromatic ring in the structure of biocides leads to increased antimicrobial activity; analogs with higher hydrophobicity have the possibility of breaking the cell membrane.	n.i.	[71]
PAA (peracetic acid) PFA (performic acid)	Amp^R^ *E. coli*	MIC 80 and 60 mg min/L 10 and 15 mg min/L	Oxidation of sulfur and sulfhydryl bonds in proteins and enzymes; disruption/dislodging the cell walls and modifying the cytoplasmic membrane of lipoproteins, blocked enzymatic and transport processes; formation of hydroxyl peroxide free radicals.	n.i.	[74]
PMA (permaleic acid)	*E. coli* ATCC 25922	MIC 40 mg/L (50%) 100 mg/L (100%)	Damage and disruption of cell membrane activity.	n.i.	[75]
ECASs (1%, 5%, 10%) (electrochemically activated solutions)	*E. coli* from the water disruption network of NUST		Oxidizing agent; induced damage to the cell membrane; Cl in ECASs increased cell permeability and disrupted protein synthesis; degradation of functional groups (ClO_2_, H_2_O_2_, ozone) over time weakens oxidative stress.	n.i.	[72]
AuNSps (nanosphere) AuNSts (nanostars) AuNCs (nanocubes)	*E. coli* from Industrial Culture Collection, China	MIC 80 µL; MBCs AuNSps—0.02 and 0.04 µg; AuNCs—0.2 and 0.4 µg	Visible surface damage, disturbance and cell loss by disruption of membrane-bound components, loss of flagella, loss of cell integrity, leakage of cell contents into the environment, death.	n.i.	[76]
Ag-iNPs	Homogenized microbial solution of *E. coli* commercial lyophilized pellet (ATCC)		Affected phospholipids, cytoplasm proteins (GADPH). Created holes on the outer membrane (OM), increased the permeability of the membrane, led to disruption of the breathing cycle leading to its lysis; electrostatic interaction, leading to disruption of the integrity of the OM and activation of OMPLA lipolytic enzymes; ROS formation	↑*PldA* ↑*cueO* ↑*copA* ↑*cusR*	[77,78]
AgNO_3_				↓*ZntA* gene ↓*CopA* gene ↓*CueO* gene	[77]
Cu/SHfNP	*E. coli* SE4 isolated from hospital wastewaters	MIC 2 mM (98%); 1 mM (91.9%)—reduced biofilm formation	Loss of cellular components of cells; reduced the level of attachment of biofilm cells; disrupted nanowire formation.	n.i.	[79]
BDCA-RNM (combination of benzophenone tetracarboxylic dianhydride and chlorogenic acid membranes)	*E. coli* O157:H7	MBC 10 µL	ROS production; cellular deformation and surface collapse; lysed and disrupted the bacterial cell walls and membranes; leakage of nucleic acids and proteins.	n.i.	[80]

↑ Upregulated ↓ Downregulated.

The current progress in nanotechnology should allow the development of novel biocides to become the most exciting new field for researchers. Such novel developments include the gold-based biocide nanomaterials (nanospheres, nanostars, and nanocubes) [76]. According to the latest studies, synthesized gold nanocubes (AuNCs) at low concentrations (0.02 and 0.04 µg) induce 100% inactivation against *E. coli*, *P. aeruginosa*, and *S. aureus* after only 30 min incubation [76]. The possible mechanisms of bacterial inactivation seem to be correlated with the physical mutilation of bacterial cells leading to cellular membrane damage and interaction with membrane-bound constituents. Similarly, silver-immobilized nanoparticles (Ag-iNPs) cause a bactericidal effect against *E. coli* [77] by upregulating the *Pld*A gene encoding a membrane phospholipase A, which is connected with the primary step of Ag-iNPs’ bactericidal mechanism that eventually triggers the appearance of breaches in the outer membrane and stresses the cellular respiration processes. More detail on the biocides’ mechanisms of action against *E. coli* is presented in Table 1.

### 4.2. Pseudomonas spp.

*Pseudomonas aeruginosa,* another member of the ESKAPE pathogen group, is well known for being able to cause serious infections due to its capacity to form highly resilient and tolerant biofilms and fight many antimicrobial agents [81]. As such it is a resistant and ubiquitous multidrug pathogen associated with many serious diseases, such as lung infections in cystic fibrosis patients and various septic syndromes [81,82]. Resistance in *P. aeruginosa* occurs due to the upregulation of efflux pumps, chromosomal β-lactamase, and mutations of antibiotic target molecules [82]. There is then no surprise that testing the efficiency of novel biocides is discussed in the scientific literature regarding their capacity to prevent biofilm formation of the most common species, including *P. aeruginosa* and *P. fluorescens*. For example, in order to combat *Pseudomonas* spp., surface contamination biocides such as the peracetic acid (PAA), sodium hypochlorite (SH), and chlorhexidine gluconate (CHDN) were tested against 185 *Pseudomonas* spp. isolates from a processing plant, with several of them demonstrating proteolytic and lipolytic activity [83]. After biofilm formation by *P. fluorescens* and *P. aeruginosa* on the stainless steel surfaces, these biocides were applied in different concentrations (SH at 100 mg/l, PAA at 300 mg/l, and CHDN at 400 mg/lL), and the results showed a statistically significant reduction in *P. aeruginosa* biofilms. The authors concluded that all three studied biocidal agents did not completely eradicate biofilms from stainless steel surfaces.

The recently designed pyridoxine-based quaternary ammonium derivatives of terbinafine, containing the active compound KFU-127, were able to significantly inhibit (at 64 μg/mL concentration) the growth of *P. aeruginosa* and partly eradicate biofilm formation [11]. Unfortunately, although this biocide has shown exceptional results in the control of other pathogens, it was also mentioned by the authors to exhibit toxic effects on eukaryotic cells, so its application should be limited. On contrary, a new biocide synthesized from soybean oil and designed to control harmful aquatic microorganisms [84] (named LGPcide) showed strong bactericidal efficiency against *P. fluorescens* and other marine microorganisms. The authors concluded that after 24 h, LGPcide reduced pathogen presence by 99.5 % and showed a low toxicity compared to other maritime industrial biocides such as menadione, zinc sulfate, diuron, and irgarol. The bactericidal effect was suggested to depend on the biocide dosage but still involved the ability to alter the cell membranes, cell fluidity, and structure.

Glycolic acid (GA) and glyoxal (GO), in contrast to reference biocides benzalkonium chloride (BAC) and peracetic acid (PAA), were used to emphasize the importance of an efficient interaction with cell membranes, in a food spoilage model, by measuring their antimicrobial activity. Using this model, the biocides were shown to interact chemically with the cell surface of *P. fluorescens* (ATCC 13525), with the exception of GO which was actually shown to physiochemically interact with bacteria. Increased doses of all biocides (PAA, BAC, GA, and GO) affected the cell culturability, and the total eradication occurred at the elevated concentrations between 0.5 and 15 µg/mL that caused a 5 log CFU/mL reduction. Glycolic acid (GA) raised the hydrophobicity and depleted the electron donor properties of *P. fluorescens* [85]. Conclusively, such biocides express their antimicrobial action by acting as a membrane-active and oxidant agent and inhibit bacterial replication. More detail on the biocides’ mechanisms of action against *Pseudomonas* spp. is presented in Table 2.

**Table 2:** Biocides and their impact on *Pseudomonas* spp.

Biocide	Strain	Concentration	Mechanism/Notes	Gene/Protein	Ref.
Cellulose foam filter paper with GA and BTCA	*P. aeruginosa* NCDC 105	MIC 1 g/L	BTCA—crosslinking agent. Possible mechanism—its acidic nature reduces the pH of the medium, which inhibits bacterial growth; Glutaraldehyde—cross-binding of the molecule with the amines of bacterial cells, interfering in the transport and enzymatic activities, which disrupts the work of the main functions, causing bactericidal effect.	n.i.	[13]
AuNSps AuNSts AuNCs	*P. aeruginosa* obtained from the China Center for Industrial Culture Collection	MIC: 80 µL; MBCs: AuNSps—0.02 and 0.04 µg; AuNCs—0.2 and 0.4 µg	Surface damage, disturbance, cell loss by disruption of membrane-bound components, loss of flagella, leakage of cell contents into the environment.	n.i.	[76]
KFU-127	*P. aeruginosa* ATCC 27853	MIC/MBC 64 µg/mL	Cell membrane damage and changes; inhibitory property on pyroxidal-dependent enzymes.	n.i.	[11]
CHX—1% BKC—1% Kohrsolin extra SEPTI-Turbo	*P. aeruginosa* NCTC 10662 and *P. aeruginosa* isolates collected from different hospitals in Hamadan city, Iran	MICs: CHX—8–128 µg/mL; BKC—8–64 µg/mL; Kohrsolin extra—8–32 µg/mL; SEPTI-Turbo—8–128 µg/mL	n.i.	↓ *cepA* gene ↓ *qacEΔ1* gene ↓ *qacE* gene	[86]
PHMG-Cl	*P. aeruginosa* ATCC 27853		PHMG-Cl attaches high molecular weight DNA and plasmid DNA, resumes the process of inactivation of DNA from surfaces.	*↓ eDNA* gene	[87]
PAA SH CHDN	*P. aeruginosa and P. fluorescens* isolated from a cheese processing line at a dairy industry located in Sao Paulo, Brazil	MBCs: PAA—300 mg/L; SH—100 mg/L; CHDN—400 mg/L	PAA—bactericidal action linked to hypochlorous acid, crosses the cell membrane, oxidizes the sulfhydryl groups of certain enzymes; SH—strong oxidizing agent of the cytoplasmic membrane, deactivating physiological functions; CHDN—reacts with the negatively charged microbial cell surface, destroying the cell membrane, penetrates into the cell and causes leakage of intracellular components.	n.i.	[83]
LGPcide	*P. fluorescens* isolated from natural seawater from Praia dos Anjos Bay, Brazil	MBC reduction >99.5% after 24 h	High concentrations cause damage to cell membranes; disturbance of membrane structure and fluidity.	n.i.	[84]
GA GO	*P. fluorescens*	MBCs: GA—1000 µg/mL; GO—15000 µg/mL	GA—active membrane and oxidizing agent, reversible on the cell envelope if applied in low concentrations; GO—effects absent on bacterial surface, cell replication inhibitor (irreversible effects).	n.i.	[85]
ILs	*P. aeruginosa* PAO1 and PA14		Permeabilization and disrupting the bacterial OM; interact with the lipid portion of the phospholipid, destabilizing the bilayer by breaking hydrophobic interaction between lipids responsible for the integrity of the membrane.	n.i.	[88]

↑ Upregulated ↓ Downregulated.

### 4.3. Klebsiella spp.

Identified in the 1880s, this pathogen is considered opportunistic in humans, being responsible for causing urinary tract and hospital-based infections, acute liver abscesses, and pneumonia [89]. Other important members of the ESKAPE pathogen group [90], *Klebsiella* spp. are frequently isolated from water and soil and are reported as capable of forming biofilm and infecting a wide variety of plants and mammals (Table 3) [91]. Recent in vitro studies indicated that *K. pneumoniae* biofilm formation can be controlled by biocidic agents such as chlorhexidine digluconate (CHX), triclosan (TRI), and ethanol (ETH) [92]. These biocides were found responsible for a significant reduction in conjugation frequency of the OXA-48 plasmids, responsible for carbapenemase production, especially in high-risk pathogenic clones. Similar to *Pseudomonas* spp., major attention is given to the efficacy of these biocides against *Klebsiella* spp. and to the possibility of resistance development. In a study from a Peruvian hospital, it was reported that *K. pneumoniae* strains pretreated with two hospital-used biocides recorded MICs from 8 to 128 µg/mL for chlorhexidine and variations from 16 to 256 mg/mL for isopropanol, highlighting an increased resistance pattern [93]. However, the biocidal activity was impacted by the temperature and growth medium, suggesting that these variables could significantly affect the biological activity of many types of studied biocides.

New biocidal products such as metal ion solutions (e.g., copper, gold, palladium, platinum, and silver) might also impact the growth and biofilm formation of K. pneumoniae [94]. A recent study has reported an effective antimicrobial metal ion solution against *K. pneumoniae* (NCTC9633), which was achieved by platinum with an MIC of 3.90 mg/L. A higher bactericidal effect was reported after pretreating bacteria with palladium, platinum, and gold at 3.90 mg/L [94]. They are also reported as having a synergistic action where elevated concentrations of metal ions resulted in total inhibition of *K. pneumoniae* biofilm formation (at 500 mg/L) by combining gold with palladium, gold with platinum, platinum with palladium, silver with palladium, or copper with gold. To prevent water contamination, it has been described that cellulose filter paper pretreated with cationic polyacrylamide and infused with glutaraldehyde solution or 1,2,3,4-butane tetracarboxylic acid (BTCA) inhibited *K. pneumoniae* (NCDC 138) growth in drinking water [13]. Metal-based nanoparticles were described as highly efficient in combating bacterial contamination in drinking water. Calcium hypochlorite (Ca(OCl)_2_), silver nanoparticles (AgNPs), and Ca(OCl)_2_/AgNPs composites showed efficient biocidal properties against *K. pneumoniae* and other pathogens isolated from tap and hand pump waters. Results have shown that AgNPs loaded with Ca(OCl)_2_ had lethal effects at the concentration of 1.5 mg/L against *K. pneumoniae*, *E. coli*, and *S. aureus* after only 180 min of exposure [95]. Conclusively, the development of impregnated filters with such metal-based nanoparticles could induce absolute biocidal effects against water-based pathogens.

**Table 3:** Biocides and their impact on *Klebsiella* spp.

Biocide	Strain	Concentration	Mechanism/Notes	Gene/Protein	Ref.
Chlorhexidine Octenidine	*K. pneumoniae* MGH 78578	MICs: 8 mg/L and 1 mg/L; MBCs: 128 mg/L and 16 mg/L	n.i.	↑ *smvA* ↑ *smvR*	[96]
Chlorhexidine Isopropanol	*Klebsiella spp.* from collections of the Universidad Cientifica de Sur in Lima, Peru. *K. pneumoniae* from HN2M, INMP, and HNGAI, Lima, Peru	MICs: 32 µg/mL (MIC_50_) and 64 µg/mL (MIC_90_)	n.i.		[93]
Derdevice plus Y I&D Sept	*K. pneumoniae* CRKP isolates obtained from Gazi University, collection of the Microbiology Laboratory	MBC 1/300 dilution and 100%	n.i.		[97]
Au, Cu, Pt, Pd, Ag	*K. pneumoniae* NCTC9633	MICs: Au—5.85 mg/L; Cu—15.62 mg/L; Pt—3.90 mg/L; Pd—5.85 mg/L; Ag—11.71 mg/L; MBCs: Au—3.90 mg/L; Cu—15.62 mg/L; Pt—3.90 mg/L; Au—3.90 mg/L; Pd—3.90 mg/L	Toxic effects of metals can cause DNA damage, antioxidant depletion, disturbance of membrane function, structural changes in the cell wall, increased cell permeability, lysis of the cell.	n.i.	[94]
Cellulose foam paper with GA and BTCA	*K. pneumoniae* NCDC 138	MIC 1–2 g/L	BTCA—crosslinking agent, reduces pH of the medium and inhibits bacterial growth; GA—cross-binding of the molecule with OM of bacterial cells interferes in the enzymatic activities and transport of the bacterial cell.	n.i.	[13]
Filter paper with Ca(OCl)_2_/AgNPs	*K. pneumoniae* isolated from different water supplies	MBC 2.0 mg/L (70%) 1.0 mg/L (50%)	Ag^+^ ions can bind and penetrate the cell membrane, increasing permeability.	n.i.	[95]

↑ Upregulated.

### 4.4. Staphylococcus spp.

*Staphylococcus* is one of the significant pathogens to be controlled in the meat industry and health establishments, particularly for its affinity to adhere the different surfaces [22]. Members of the genus *Staphylococcus* can colonize the skin and airways of animals and birds, while highly pathogenic strains are associated with the events of minor, severe, or fulminant infections. To date, through unpredictable evolutionary processes, some of *Staphylococcus* species being ordinary constituents of human/animal microbiota have shifted towards evolving in pathogen-progressive strains [22].

Biocide resistance is a major issue in *S. aureus*, and preventing such resistance is a significant challenge for scientists. Human-associated *S. aureus* strains were reported to harbor a specific bacteriophage ΦSa3 which encrypts tricky evasive immune factors, but more recently, this prophage has also been detected in livestock-distributed *S. aureus* (MRSA) CC398 and evolved as a facilitator of human colonization [98]. In earlier studies, the ancestor of *S. aureus* (CC398) was reported as a strain adapted to humans, but due to the extensive human–livestock activities, it was transferred to animals via the loss of prophage Sa3 [99]. Commercial biocides based on hydrogen peroxide were recently described as capable of inducing the phage Φ13 from the Sa3 group that encodes human immune evasion genes associated with human colonization [98]. Furthermore, in vitro results showed that sublethal concentrations of hydrogen peroxide or biocides based on hydrogen peroxide were responsible for the transfer of prophage Φ13 from human *S. aureus* donor strain to livestock-associated MRSA CC398 61599 recipient strain. Clinically relevant isolates (MRSA and MSSA) can encode biocide resistance genes such as *qac*A/B and *qac*C [100]. An illustration of biocide resistance was described in *S. aureus* strains isolated from clinical samples from Egypt and showed that the presence of qacA/B genes caused increased MICs of sodium hypochlorite, phenol, and Savlon (chlorhexidine digluconate 0.3% and cetrimide 3%). Likewise, the isolates of MRSA sampled from infected and healthy humans and animals from Germany recently displayed the presence of biocide and heavy metal tolerance-mediating factors such as *cop*A, *czr*C, *lmr*S, *mco*, *mep*A, *nor*A, *qac*A, *qac*G, *qac*J, *sep*A, and *smr*. Moreover, some of the important human lineages (e.g., CC22 and CC5) illustrated statistically significantly greater MICs for acriflavine, benzethonium chloride, and chlorhexidine compared with the origin of animal sequence type 398 (ST398) [101].

Most biofilm studies concentrate on the mechanistic aspects of biocide resistance. The promising biocidal nanoparticles [102] based on thymol-loaded chitosan silver nanoparticles (TCAgNPs) versus the biofilm formation and virulence-associated proteins of *S. aureus* (Bap-MRSA 090) had the capacity to reduce biofilm formation in a dose-dependent manner, with the most promising activity seen at the concentration of 200 µg/mL causing a significant 73.37% reduction compared to controls. Moreover, the higher concentrations (500 µg/mL) of TCAgNPs after 48 h exposure to *S. aureus* repressed the expression of *Coa*, *Eap*, and *SpA* exoprotein genes in contrast to the housekeeping control gene. It can be concluded that such approaches based on nanoparticles can specifically trigger and deactivate biofilm formation and impair the cell membrane of immune-evasive MRSA strains at MICs of only 1 mg/mL [103]. An innovative approach reported that iron oxide nanoparticles transported within the matrix through a magnetic field against MRSA can result in biofilm inactivation [104]. After the MRSA biofilm generation, the treatment with 5 mg/mL of magnetic nanoparticles (e.g., spheres, cubes, and tetrapods) in a rotating magnetic field condition induced significant differences and was achieved by cubic and tetrapod nanoparticles that displayed a 14.19 log10 CFU/plate eradication, while spheric nanoparticles displayed only a 7.3 ± 0.2 log10 decrease. Given the large amount of data currently present in the literature, we have summarized the main results in Table 4.

**Table 4:** Biocides and their impact on *Staphylococcus* spp.

Biocide	Strain	Concentration	Mechanism/Notes	Gene/Protein	Ref.
Benzalkonium chloride, H_2_O_2,_ Biocide 1, Biocide 2, Biocide 3, mitomycin C	*S. aureus* 8325-4 and phages 8325-4Φ13 (CG1); RN420 8325-4Φ13-kana; MW2-ΦSa3mw; MW2c; 61599; 93616; DC10B; 8325-4Φ13attBmut; RN4220Φ13attBmut	MICs: BC—2.67 µg/mL; H_2_O_2_—0.03% w/w; Biocide 1—0.02%; Biocide 2—0.02%; Biocide 3—5.00 %; mitomycin C—0.12 mg/L	Known as *hlb*-converting phages which integrate in the *hlb* gene at the *att*B attachment site.	n.i.	[98]
Chlorhexidine	*S. aureus* isolates ST36 (*qacA^+^* and *qacA^−^*); ST22 (*qacA^+^* and *qacA^−^*); ST239-TW (*qacA^+^*)	ST22 clones—decrease 45% (1.47 mg/L); ST36 clones—1.75 mg/L	Increased binding to fibrinogen and fibronectin, increased adhesion and internalization into monolayers of keratinocytes, and confirms phenomenon of survival in vivo after chlorhexidine exposure.	n.i.	[105]
Hypochlorite and Phenol	MRSA and MSSA *S. aureus* clinical isolates, Egypt	Association between the presence of antiseptic resistance genes and the MICs	n.i.	↑*qacA/B*, *qacC* genes	[100]
Chlorhexidine	201 MRSA isolates from Portuguese hospitals, strain collection at ITQB-NOVA in Oeiras, Portugal	MICs 0.125–4 mg/L, one of them presented MICs 0.5–1 mg/L MBCs 0.125–8 mg/L	n.i.	*sepA* and *mepA (100%); lmrS* (87.1%)*; qacAB (22.4%); smr (1.0%)*	[106]
F10SC Hexacon	*S. pseudintermedius* (MRSP, MSSP) and *S. aureus* (MRSA) clinical isolates, Sydney, Australia	MBCs: F10SC—1.05–16.87 mg/L; Hexacon—7.81–31.25 mg/L	n.i.	*qacA/B* genes—were only in MRSA isolates. *qacJ* (54%*), qacG* (29%), *smr* (7%) MRSP isolates.	[107]
Cu/SHfNP	*S. aureus* ATCC 6538	Reduced cell growth at the highest concentration (1 mM) by 86%	Damage cell membrane, increase membrane’s permeability, disrupt cell membrane at high dose (2 mM).	n.i.	[79]
Dex-MA	*S. aureus* (MRSA) (ATCC 33591)	MICs: 1–2 µg/mL; MBCs: Dex-5—1.0 mg, Dex-10—1.9 mg, Dex-20—4.1 mg	n.i.	n.i.	[108]
PAA Chlorhexidine digluconate	*S. aureus* BZ012 and Sa30 from Brazilian dairies	MICs: PAA—0.075% (4.6 log) and 0.015% (1.1 log) mixed cultures; chlorhexidine dicluconate—0.0001953125 and 0.025% mixed cultures.	n.i.	n.i.	[109]
T-C@AgNPs	*S. aureus* (Bap-MRSA)	n.i.	Damage and destabilization of membrane of bacterial cell. Bioelectrical changes caused by biocide (intramembrane space to outside the cell) create pores—dose-dependent.	↓*Coa*, ↓*Eap*, ↓*SpA*, ↓*Bap genes*	[102]
GTAgNPs	*S. aureus* 090 (MRSA090)	MIC 20 mg/mL	Presents antioxidant dose-dependent activity. Neutralizing ROS.	↓*Coa*, ↓*Eap*, ↓*SpA* genes	[103]
PVDF/GN and PVDF/SP	*S. aureus* ATCC 25923	Caused a 6-log reduction	Bactericidal action confirmed with the presence of intracellular ROS.	n.i.	[12]
MBQ (magnetic biochar/quaternary phosphonium salt)	*S. aureus* ATCC 6538	MBC: 2 mg/L (50%), 50 mg/L (90%), dose-dependent effects	Loss of cell integrity, the appearance of vacuolization, rupture of cell surface and leakage of intracellular substances, Induced oxidative stress, penetrates through the lipid bilayers, and increases membrane structural destabilization.	n.i.	[73]
AuNSps AuNSts AuNCs	*S. aureus* from CICC, Beijing China	MICs 0.04 µg	Surface damage, disturbance, and cell loss by disruption of membrane-bound components, loss of flagella, cell integrity, leakage of cell contents into environment.	n.i.	[76]
CTAB	*S. aureus (MRSA)*	Cubic and tetrapod nanoparticles—14.19 log_10_ CFU/plate eradication; spheric nanoparticles—7.3 ± 0.2 log_10_ decrease	n.i.	n.i.	[104]
KFU-127	*S. aureus* ATCC 29213, *S. epidermidis* clinical isolate from Kazan Institute, Russia	MICs: 4 µg/mL; MBCs: 8 µg/mL	Membrane potential changes and cell membrane damage; inhibitory property on pyroxidal-dependent enzymes.	n.i.	[11]

↑ Upregulated ↓ Downregulated.

### 4.5. Listeria spp.

*Listeria* spp. are rod-shaped, Gram-positive bacteria commonly found in assorted food products and on industrial surfaces; they are notorious for causing life-threatening infections known as listeriosis and septicemia [22,110]. Several outbreaks were associated with the consumption of contaminated dairy and ready-to-eat (RTE) products, including fruit and vegetables [111]. Biocide tolerance in *L. monocytogenes* strains (Table 5) shares similar mechanisms to antibiotic tolerance [112]. However, some authors did not identify biocide and antibiotic cross-resistance in their in vitro adaptation experiments when bacteria were exposed to sublethal concentrations of disinfectants. [113]. However, others indicated that strains isolated from diverse ecological niches exposed to quaternary ammonium compounds (QACs) coselected antibiotic resistance. According to the same authors, *Listeria* spp. have the ability to adapt to biocides, especially QACs, and adaptation is linked to coresistance to ciprofloxacin [114].

Inappropriate use of disinfectants could result in acquired resistance, and it was previously illustrated that *L. monocytogenes* could become resistant to biocides (benzalkonium chloride) and cause improved biofilm formation in BZK-resistant strains by involving various resistance mechanisms and genetic mechanisms (Table 5) [121]. *Listeria* strains from Brazilian tilapia-processing facilities are examples of such resistant strains as they had significantly increased ability to form biofilm on polystyrene and stainless steel surfaces [122]. Higher concentrations of sodium hypochlorite and peracetic acid were required for complete eradication, indicating an increased resistance. The biofilm formed on polystyrene by *L. monocytogenes* isolates, from pork meat, was significantly reduced when pretreated for 10 min with sodium hypochlorite (MICs ranging from 1 to 1.5) and benzalkonium chloride ((MICs ranging from 0.5 to 1.5) [123]. The maximum reduction of 90% was reported for sodium hypochlorite. In conclusion, none of the biocides were able to induce a complete eradication of biofilm. The decreased eradication capacity of benzalkonium chloride is caused by the presence of resistance determinants bearing *bcr*ABC and *qac*H genes [124,125]. A similar pattern was described for *L. monocytogenes* isolates from food-processing environments in southern Brazil harboring the efflux pumps *Mdr*L and *Lde* and resistance to cadmium chloride [120]. Separate review papers are necessary to link the ability of a microorganism to multiply with its resistance capacity as resistance might provide certain pathogens with the necessary ability to inhibit the growth of other bacteria for their own benefit.

### 4.6. Campylobacter spp.

*Campylobacter* spp. are a spiral-shaped, Gram-negative, microaerophilic bacteria being especially more prevalent in poultry than in ubiquitous environments [20]. These bacteria have a unique fast transmissibility and colonization rate in animals and humans, predominantly via consumption of contaminated water, food, eggs, milk, and undercooked meat. On a global scale, *Campylobacter* is the major cause of gastroenteritis and is one of the most widespread causative pathogens of infectious diseases of the last century [126]. As such, controlling the spread of this pathogen is vital, not only in food processing facilities, but also in hospital or poultry farming environments and solutions are provided in Table 6. A recent article indicated that 99% of poultry-isolated *C. jejuni* strains had highly increased resistance to triclosan, 32% of strains showed resistance to chlorhexidine, and all of the strains were susceptible to benzalkonium chloride [127]. The most effective antimicrobial substance was didecyldimethylammonium chloride, which also acted in a synergetic way with benzyldimethyldodecylammonium chloride and inhibited *C. jejuni* [128].

*Campylobacter* is indeed a serious and persistent issue for the poultry industry and identification of efficient biocides is still a struggle [132]. Chlorine, one example, is widely used as a standard biocide in the poultry industry (Table 6) to decrease bacterial loads in poultry carcasses and skins. Recent in vitro research showed that chlorine might effectively decrease *C. jejuni* in poultry carcasses before washing, but notably without completely eradicating the pathogen [129]. The complete pathogen inhibition occurred at chlorine MICs above 8 ppm. However, due to genetic variations of the species and different involved tolerance mechanisms, chlorine sensitivity can vary in *Campylobacter*. In some cases, at high bacterial growth concentrations, *Campylobacter* spp. were resuscitated after chlorine treatment, suggesting an update of chlorine usage or new possible alternatives. More promising effects were achieved in another in one of our studies in which an antimicrobial mixture based on sodium chloride, sodium hydroxide, citrus, oregano, and grape seed extracts reduced pathogen presence on poultry neck skins and whole carcasses [20]. On the other hand, in healthcare facilities, puroindoline A (PinA) could act as a therapeutic biocide or as a preservative agent in the agri-food industry [130]. In vitro analysis has reported that PinA peptides inhibited *C. jejuni* (81-176 and F38011) biofilm formation at 256 µg/mL concentration. Treatment of *C. jejuni* isolates with PinA disrupted and modified the cell membrane and made it prone to permeability to other external substances. The PinA mechanism of membrane disruption via the formation of pores in the lipid bilayers was interlinked with the existence of disulfide bonds and hydrophobic tryptophan-rich domains (TRDs) in its chemical structure.

### 4.7. Salmonella spp.

The clinical manifestations of *Salmonella* infections are similar with those of *Campylobacter* infections, with both requiring the use of antibiotics and proton pump inhibitors to reduce symptom severity [22]. According to the latest data, salmonellosis is the second most documented gastrointestinal human infection in Europe after campylobacteriosis [133]. Furthermore, *Salmonella* can form robust biofilms on various abiotic and biotic surfaces, which confers tolerance to several stresses and antimicrobials, including biocides [134]. Biocides aiming to be efficient in eradicating *Salmonella* in food processing environments will have to overcome the increased expression of efflux pump genes and its high capacity for biofilm formation. It was suggested that biofilm-forming bacteria must also be considered for complete and efficient sanitation of food-contact surfaces besides the planktonic cell targeting [135]. The biological mechanisms involved in the eradication of *Salmonella* spp. by biocides are as complex as those for the other microorganisms discussed in this review (Table 7).

Novel biocides, such as the black cardamom essential oil, inhibit the biofilm formation in *S. Typhimurium* JSG 1748, by approximately more than 50%, at concentrations from 0.03 to 0.5% when they inhibit the quorum-sensing mechanisms of biofilm formation [139]. Other essential oils, such as thymol, were also highlighted to express better antibiofilm potential than benzalkonium chloride against *Salmonella enterica* serovar *Typhimurium* DT193 strain [136] at a concentration of 1250 ppm. However, their application on the stainless steel coupons resulted in a 5-log reduction compared to the initial growth inoculum.

## 5. Conclusions

The findings of this review conveyed the broad antibacterial, antiadhesive, and antibiofilm capabilities of various biocidal agents against emerging dangerous pathogenic bacteria. These data could play a lasting indicative role soon due to the description of competent disinfection strategies presented herein. Proper disinfection is a key element of successfully inactivating bacterial and viral pathogens in different agricultural, medical, and agricultural facilities, including the equipment, utensils, and surfaces that urgently require sterilization under the existing pandemic circumstances. The biocontrol measures described in this review emphasize firstly the importance of novel strategies aiming to avoid antibiotic–biocide cross-resistance in pathogenic agents and secondly the necessity of understanding the molecular mechanisms involved.

## Figures and Tables

**Table 5:** Biocides and their impact on *Listeria* spp.

Biocide	Strain	Concentration	Mechanism/Notes	Gene/Protein	Ref.
Benzalkonium chloride	*L. monocytogenes* SLCC2540	MIC 4 µg/mL; MBC 11 µg/mL	Increased abundance of viable but nonculturable cells.	May induce mutations in efflux pump systems, which are responsible for multidrug resistance	[115]
	*L. monocytogenes* ST6	Greater than 10 μg /mL	n.i.	BC tolerance due to the presence of pLMST6 plasmid (*EmrC* gene responsible for the multidrug efflux pump protein)	[116]
	*L. monocytogenes* isolated from South Africa food chain (e.g., ST1, ST2, ST121, ST204, and ST321)	n.i.	n.i.	↑*bcrABC* cassette ↑*ermC* ↑*mdrL* ↑*Ide* ↑SSI-1 ↑ SSI-2	[117]
BP1-BP5BP6-BP11	*L. monocytogenes* isolated strains 2075, 2081, and 2355 from the mushroom production environment. Strains 3050, 3051, 3101, 3102, and 3104 isolated from the production environment after steam cookout	1.7-log and 6-log reductions; the highest reduction, 6-log—BP6 and BP11 MICs ranged between 1 and 50%	n.i.	n.i.	[118]
Triclosan	*L. monocytogenes* *L. welshimeri*	MIC from 0.0015 to 0.006%	n.i.	n.i.	[119]
Cadmium chloride	*L. monocytogenes* isolates from food-processing environments, Brazil	Ranged from 20 to 200 µg.mL^−1^	n.i.	Presence of the efflux pumps MdrL and Lde, cadmium chloride resistance	[120]
PAA Chlorhexidine	*Listeria* monocytogenes BZ001 isolated from a single food-processing environment	MIC 0.075% for PAA and 0.000390625% for chlorhexidine	n.i.	n.i.	[109]

↑ Upregulated.

**Table 6:** Biocides and their impact on *Campylobacter* spp.

Biocide	Strain	Concentration	Mechanism/Notes	Gene/Protein	Ref.
Triclosan Chlorhexidine Benzalkonium chloride	96 *C. jejuni* strains isolated from poultry industry	MIC50 = 32 and MIC90 = 32 (μg/mL); MIC50 = 0.5 and MIC90 = 1; MIC50 = 1 and MIC90 = 2	n.i.	99% of strains showed triclosan resistance; 32% chlorhexidine resistance; 100% susceptible to benzalkonium chloride	[127]
Chlorine	*C. jejuni* from poultry carcasses	MIC: 8 ppm for 97.5%; MBC: 128 ppm	Dose-dependent mechanisms, can be resuscitated after chlorine treatment.	n.i.	[129]
Antimicrobial mixtures based on sodium chloride, sodium hydroxide, citrus, oregano, and grape seed extracts	*C. coli* isolates (NC1, NC2, and NC3) and cecum strain (RC018)	MIC: 0.25–1%	n.i.	↓ inhibit T6SS (hcp)	[20]
PinA	*C. jejuni* (81-176 and F38011)	MIC: 256 µg/mL	Membrane disruption, pore formation, increases membrane permeability.	n.i.	[130]
Disinfectant based on glutaraldehyde, formaldehyde, and antifreeze	*C. fetus* subsp. venerealis and *C. fetus* subsp. Fetus	MIC: 0.5%	n.i.	n.i.	[131]

↓ Downregulated.

**Table 7:** Biocides and their impact on *Salmonella* spp.

Biocide	Strain	Concentration	Mechanism/Notes	Gene/Protein	Refs.
Chlorine QACs	*S. enterica* isolated from processing environments	Chlorine—500–1000 ppm QACs—3–25 ppm	n.i.		[135]
Thymol	*S. enterica* serovar *Typhimurium* epidemic phage type DT193 strain	1250 ppm total eradication	n.i.		[136]
Ozonated water	*S. Heidelberg* multidrug-resistant isolated from pigs (farms)	MIC 4.4 mg/L	Induces changes in bacterial DNA after 10 min exposure.	n.i.	[137]
Sodium hypochlorite PAA Chlorhexidine	*S. enterica* serovar *Minnesota* strains isolated from a poultry slaughter, Brazilia	1% SH (100%), 0.8% PAA, and 1% chlorhexidine decrease bacterial counts by 3.63 and 2.96 CFU/mL	n.i.	Highly virulent due to factors: avrA, agfA, lpfA, sodC, and luxS genes	[138]
